# Complete Genome Sequence of a Clinical *Campylobacter* Isolate Identical to a Novel *Campylobacter* Species

**DOI:** 10.1128/MRA.00721-20

**Published:** 2021-02-18

**Authors:** Birgitta Duim, Linda van der Graaf-van Bloois, Arjen Timmerman, Jaap A. Wagenaar, Jacky Flipse, Janny Wallinga, Peter Bloembergen, William G. Miller, Aldert L. Zomer

**Affiliations:** a Faculty of Veterinary Medicine, Department of Infectious Diseases and Immunology, Utrecht University, Utrecht, the Netherlands; b WHO Collaborating Center for Campylobacter/OIE Reference Laboratory for Campylobacteriosis, Utrecht, the Netherlands; c Wageningen Bioveterinary Research, Lelystad, the Netherlands; d Laboratory of Medical Microbiology and Infectious Diseases, Isala Clinics, Zwolle, the Netherlands; e Produce Safety and Microbiology Research Unit, Agricultural Research Service, U.S. Department of Agriculture, Albany, California, USA; University of Maryland School of Medicine

## Abstract

Here, we present the complete genome sequence of a *Campylobacter* strain isolated in the Netherlands from a patient with gastroenteritis. The strain showed >98% sequence identity to the novel *Campylobacter* species sequence recently recovered from metagenomic data, isolated from breastfed infants with diarrheal disease, and named “*Candidatus* Campylobacter infans.”

## ANNOUNCEMENT

Here, we present the genome sequence of *Campylobacter* strain 19S00001, which was isolated from a 42-year-old male in the Netherlands with relapsing diarrhea whose stool samples were repeatedly positive by a *Campylobacter*-specific quantitative PCR (qPCR) (1, 2). To isolate this *Campylobacter* strain, 1 g of fecal material was suspended in 1.5 ml phosphate-buffered saline (PBS), and subsequently, 300 μl of this suspension was added onto a cellulose acetate membrane filter (0.65 μm; Millipore) placed on Columbia agar with 5% sheep blood (Oxoid, Thermo Scientific, Inc.) (3). After 30 min, the filter was removed and the plate was incubated under microaerobic conditions (83.3% N_2_, 7.1% CO_2_, 3.6% H_2_, and 6% O_2_) at 37°C for 72 h. One *Campylobacter*-suspected colony was detected and subcultured on Columbia agar with 5% sheep blood under microaerobic conditions at 37°C for 5 days. DNA was isolated using the DNeasy UltraClean microbial kit (Qiagen, Venlo, the Netherlands), was not sheared, and was size selected for sequencing. The Illumina library was prepared using the Nextera kit (Illumina, San Diego, CA, USA). Pooled libraries were sequenced using a NextSeq system providing 1,068,916 reads (*N*_50_ of 150 bp) that were trimmed using TrimGalore v0.4.4 (https://github.com/FelixKrueger/TrimGalore). Nanopore sequencing was performed according to protocol SQK-LSK109 with flow cell type R9.4.1 on a MinION device (FLO-MIN106D; Oxford Nanopore, Oxford, United Kingdom), using the live (fast) basecalling method in MinKNOW v19.12.1 on a MinIT device, providing 2,484,000 reads (*N*_50_ of 6.4 kb) that were trimmed and downsampled to 200× coverage using filtlong (https://github.com/rrwick/Filtlong), and resulting in 17,582 reads (*N*_50_ of 23 kb) that were assembled using metaFlye v2.7 (4) into a single scaffold. The genome was circularized and rotated using a DnaA database in Unicycler v0.4.7 (5) with overlapping Illumina and Nanopore reads. This resulted in two circular contigs, representing a 1,754.5-kb chromosome with a GC content of 35.9% and a 5,856-kb plasmid. Prokka v1.13 (6) was used for annotation with “*Candidatus* Campylobacter infans” as an additional custom database (GenBank accession number SPMW00000000.1) (7, 8). A core genome phylogeny without correction for recombination was reconstructed using FastTree (9), based on a 365,157-bp core gene superalignment of 351 core genes of which the protein sequences had at least 35% sequence identity, as determined by Roary v3.12.0 (10). The average nucleotide identity (ANI) was identified using JSpecies v1.2.1 (11). Default parameters were used for all software unless specified otherwise.

**FIG 1:**
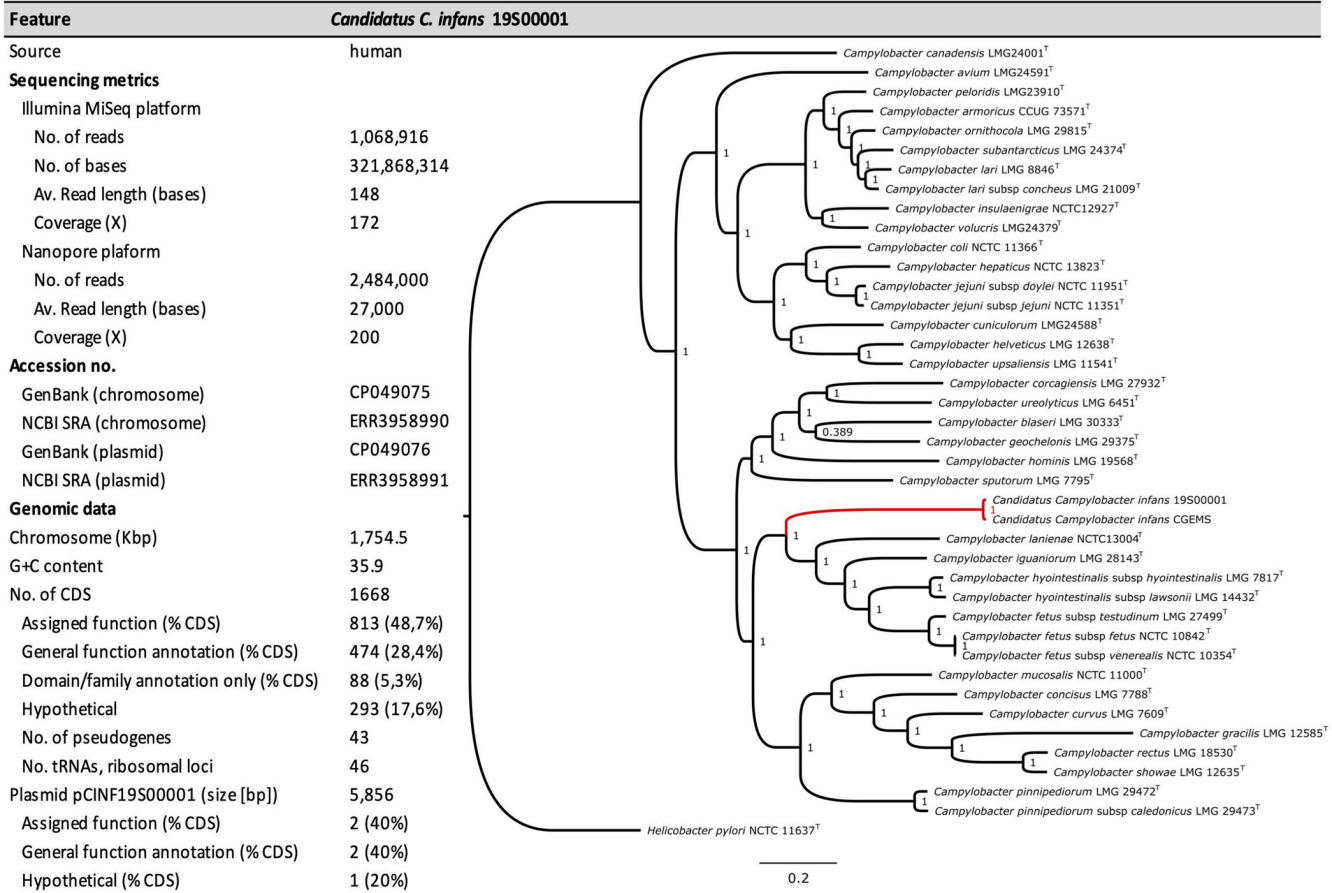
Sequencing metrics, genomic data, and phylogenetic dendrogram based on the core genome single nucleotide polymorphisms (SNPs). Helicobacter pylori J99 is used as an outgroup and root. Local support values, calculated with the Shimodaira-Hasegawa test (14), for all branches are given. Bar, 0.2 substitutions per nucleotide position in the core gene superalignment.

The sequence clustered with the genome of the recently identified novel putative species belonging to the Campylobacter fetus group “*Candidatus* Campylobacter infans” (Fig. 1) (7) with a 98.27% ANI.

The 19S00001 genome contains a type II-B CRISPR-Cas system (12). A putative integrated phage containing genes for a type IV (T4SS) conjugative transfer system (13) and a second phage integration containing a zonula occludens toxin (Zot) island were identified. The S-layer secretion system gene *sapDEF*, as well as d-arabinose 5-phosphate isomerase (*kpsF*), and flagellin-associated genes were present as identified in the genome of “*Candidatus* Campylobacter infans” (7). This study shows the first complete genome and plasmid sequences of a clinical isolate with high sequence identity to a novel *Campylobacter* species.

### Data availability.

Genome and plasmid sequences have been deposited in GenBank under the accession numbers CP049075.1 and CP049076.1. The Nanopore reads have been deposited in SRA under the accession numbers ERR3958990 and ERR3958991 (Fig. 1).
